# Glycaemic Response to Quality Protein Maize Grits

**DOI:** 10.1155/2010/697842

**Published:** 2010-08-25

**Authors:** Leonora N. Panlasigui, Cecile L. T. Bayaga, Erniel B. Barrios, Kim L. Cochon

**Affiliations:** ^1^Philippine Women's University, School of Nutrition, Taft Avenue, Manila 1004, Philippines; ^2^Department of Food Science and Nutrition, College of Home Economics, University of the Philippines, Diliman, Quezon City 1101, Philippines; ^3^School of Statistics, University of the Philippines, Diliman, Quezon City 1101, Philippines

## Abstract

*Background*. Carbohydrates have varied rates of digestion and absorption that induces different hormonal and metabolic responses in the body. Given the abundance of carbohydrate sources in the Philippines, the determination of the glycaemic index (GI) of local foods may prove beneficial in promoting health and decreasing the risk of diabetes in the country. *Methods*. The GI of Quality Protein Maize (QPM) grits, milled rice, and the mixture of these two food items were determined in ten female subjects. Using a randomized crossover design, the control bread and three test foods were given on separate occasions after an overnight fast. Blood samples were collected through finger prick at time intervals of 0, 15, 30, 45, 60, 90, and 120 min and analyzed for glucose concentrations. *Results*. The computed incremental area under the glucose response curve (IAUC) varies significantly across test foods (*P* < .0379) with the pure QPM grits yielding the lowest IAUC relative to the control by 46.38. Resulting GI values of the test foods (bootstrapped) were 80.36 (SEM 14.24), 119.78 (SEM 18.81), and 93.17 (SEM 27.27) for pure QPM grits, milled rice, and rice-QPM grits mixture, respectively. *Conclusion*. Pure QPM corn grits has a lower glycaemic response compared to milled rice and the rice-corn grits mixture, which may be related in part to differences in their dietary fibre composition and physicochemical characteristics. Pure QPM corn grits may be a more health beneficial food for diabetic and hyperlipidemic individuals.

## 1. Introduction

Carbohydrates are the main source of energy for the human body. However, the rate of digestion and absorption of carbohydrates varies with the chemical components of the food source, the processing and storage conditions it was subjected to and the other foods that were consumed in conjunction to the carbohydrate-rich food. As shown previously, even through a constant amount of available carbohydrates, significant variations may still be observed in the glucose response to different carbohydrate foods [1]. Thus, the glycaemic index (GI) has been developed to classify carbohydrate foods based on the rate of carbohydrate absorption. 

Carbohydrates that exhibit low glucose response after ingestion have been shown to be beneficial in the management of diabetes and hypelipidemia [2–4]. Given the abundance of carbohydrate-rich foods in the Philippines, knowledge of the GI may prove to be beneficial in the prevention and management of prevalent metabolic diseases, such as diabetes, in the country. However, only a few local studies have been conducted to determine the GI of local food items [5–11].

Corn is considered as a secondary staple to rice in the Philippines. According to the National Nutrition Survey conducted by FNRI-DOST last 2003, corn-eating regions in the country usually consume this cereal in the form of grits which are produced by milling white corn similar to rice. Both rice and corn are rich in carbohydrates although their functional and physicochemical properties differ. The preference towards consumption of rice stems from various cultural, economic, and nutritional factors—one of which is the inferior protein quality of common corn varieties compared to rice. The development of Quality Protein Maize (QPM), a high-breed flint corn variety that contains the amino acids, lysine and tryptophan, changes this “inadequacy” of maize. The leveling off in the protein components of rice and corn may just be the solution to the search for a better alternative to importation given the limitation in the country's rice supply. Investigating possible benefits of consuming QPM corn may give the necessary push to promote the consumption and production of this indigenous food crop.

## 2. Materials and Methods

### 2.1. Subjects

Ten apparently healthy female subjects from the College of Home Economics, University of the Philippines, Diliman, Quezon City, Philippines were selected based on the following criteria: age 18–30 years, no intake of metabolic drugs, and nonsmokers. Potential participants were contacted either through mobile phone or approached personally. Each individual was given a Subjects' Brochure, which enumerates the research objective, procedures, schedule, and other details of the study. A research staff also explained these information before each potential subject was asked for their comments and concerns on the study. Each potential subject was also interviewed for assessment of physical activity and was asked to fill in a three-day food intake recall form. Subjects with normal food intake and physical activity were included in the study. The subjects signed voluntary consent forms approved by the Department of Ethics Review Committee of the University of Santo Thomas, Manila, Philippines. 

### 2.2. Test Foods

QPM corn grits samples were obtained from the Institute of Plant Breeding (UP College of Agriculture, Los Baños, Philippines). Corn used was harvested after 65 to 70 days before it was dehusked and then sun dried for 2 to 3 days. After drying the corn samples, the dried corn kernels were mechanically removed from the cob and then sun dried for another one to two days to ensure that moisture content was less than 12 percent to inhibit fungal growth and aflatoxin contamination. Dried corn kernels were milled using a standard milling machine so that resulting total quantity of particles would amount to 30% of total weight of corn kernels processed.

Rice samples of PSB RC72H (Mestizo1 rice) variety were obtained from the Philippine Rice Research Institute (Nueva Ecija, Philippines). After aging for 123 days, samples were harvested and then dehulled with a mechanical dehusker. Afterwards it was milled in a one-pass mill to produce the white rice.

Twenty-five grams available carbohydrate portions of pure QPM grits, milled rice, and QPM grits mixture were used in the in vivo testing. These were prepared through boiling one hundred fifty grams of raw samples in water. For the pure QPM grits, the sample was soaked for 60 minutes in 325 grams of water and then boiled in the water used for soaking for a total of 35 minutes on a La Germania electric stove on medium setting for 12 minutes, then on low setting for 13 minutes, and lastly on simmer setting for 10 minutes. The 359 grams yield was divided into 117.4-gram pure QPM grits portions. On the other hand, milled rice was boiled in 240 grams of water for a total of 25 minutes on a La Germania electric stove on medium setting for 5 minutes, then on low setting for 10 minutes, and lastly on simmer setting for 10 minutes. The 350 grams yield was divided into 119.7-gram milled rice portions. Lastly, the mixture of 87 grams QPM corn grits and 58 grams milled rice variety was soaked for 30 minutes using 325 grams of water and was then boiled in the water used for soaking for a total of 35 minutes on a La Germania electric stove on medium setting for 11 minutes, on low setting for 14 minutes, and on simmer setting for 10 minutes. The 373 grams yield was divided into 85.9-gram portions of the mixture. The electric stove used was preheated on high setting for 2 minutes before cooking both test foods.

The white bread, which was used as the standard for the glycaemic index testing, was prepared based on the formulation of Panlasigui and Thompson [11] which consists of all-purpose white flour (250 grams bleached, enriched, Pilsbury brand, Pilsbury Co., Philippines), lukewarm water (150 mL), refined white sugar (7 grams), iodized salt (1.25 grams), and active dry yeast (8 grams). The bread was baked using a standard method of mixing and then kneading, fermentation (30 minutes at 40°C for the first rising of the dough and 1 hour and 340 minutes at room temperature for the second rising of the dough), and finally baking at 375°C for 20 minutes. Cooked bread is divided into 50-gram portions.

### 2.3. Protocol

Each subject was instructed to fast for 10–12 hours and refrain from any strenuous physical activity a day prior to the in vivo testing. During the test proper, the subjects were given a 10–15 minutes rest after their arrival before the fasting blood samples were obtained. The food sample assigned for the given day was taken within a 15-minute period and the subject's exact eating time was recorded. Each meal occasion was accompanied by 220–250 mL water which is made constant for each subject throughout the feeding sessions. 

Finger prick blood samples were obtained by gentle pressure at the finger tip then puncturing the skin with an autolancet (MediSense, Abbott Laboratories, Illinois, USA) at time intervals of 0 (FBG), 15, 30, 45, 60, 90, and 120 minutes through the assistance of a registered medical technologist from the UP Health Service in Diliman, Quezon City, Philippines. Approximately three to five drops of whole blood samples were collected and placed into 80 IU/ml soda lime glass microtubes that were sodiumheparinized (Vitrex, Modulohm A/S, Vasekaer 6–8, DK 2730 Herlev, Denmark). Samples were centrifuged using a Microtube Centrifuge (Vernitron Medical products, Inc., Carlsladt, New Jersey, USA) to isolate the plasma component of the blood. Ten microliters (10 *μ*L) of isolated blood plasma samples were then pipetted into previously prepared and labeled test tubes containing 1.5 mL of blank (distilled water) and standard glucose oxidase (Mega diagnostics, LA, CA, USA 90012) reagents that were incubated in a water bath incubator (Chicago surgical and electrical Co, Melrose Park, Illinois) for 5 minutes at 37°C. After the isolated blood plasma samples were pipetted, the respective tubes were mixed and again incubated for ten (10) minutes at 37°C. Blood plasma parameter was analyzed for its glucose concentration using a Dialab DTN 410 Photometer (Boehringer Mannheim GmbH, Germany) with absorbance set at 500 nm.

The area under the glucose response curve for each food was calculated geometrically [12]. The GI of each food was expressed as % mean glucose response to the test food divided by the standard food taken by the same subject and was determined using the following formula:

(1) $$\begin{array}{c} \text{GI}=\frac{\text{IAUC}\;\text{of}\;\text{the}\;\text{test}\;\text{food}\times 100,}{\text{IAUC}\;\text{of}\;\text{the}\;\text{standard}\;\text{food}} \end{array}$$

where the IAUC is the incremental area under the glucose response curve.

### 2.4. Proximate Analysis

The test foods were analyzed for total available carbohydrates (TACs), ash, moisture, crude fat, crude protein, and total dietary fibre (TDF). TAC was determined using the modified Clegg Anthrone method [13]. Ash content was determined by dry oxidation method at ≤550°C (AOAC No. 923.03). Moisture content was determined using the reduced pressure and temperature method (AOAC No. 934.01). Crude fat and crude protein were analyzed by using the solvent extraction of moisture-free samples method (AOAC No. 920.39C) and Kjeldahl method (AOAC No. 920.87), respectively. Lastly, the total dietary fiber content was determined using the enzymatic gravimetric method using MES-TRIS buffer (AOAC No. 991.43).

### 2.5. Statistical Analysis

The significance was calculated by analysis of variance (ANOVA) using Stata ver.6.0 (Stata Corporation, Texas, USA). Reanalysis of the GI values of the test food was done by regression analysis and bootstrapped method using Stata ver.6.0 (Stata Corporation, Texas, USA). Reanalysis was performed to reduce the potential bias induced by some extreme values in the data gathered.

## 3. Results

Table 1 shows the characteristics of the subjects. The ten subjects were female aged 19–22 years and had a mean BMI of 19.4 (SEM 0.6) kg/m^2^ at baseline. There were no significant changes in the subjects' anthropometric measurements at baseline and post test. 

Table 2 shows the proximate composition of the test foods. Boiled rice-QPM grits (28.92%) had the highest TAC followed by the boiled QPM corn grits (20.94%) and boiled rice (20.88%), respectively. On the hand, milled rice had the highest crude fat (3.26%), crude protein (3.14%), and moisture (72.52%).

Mean blood glucose concentration peaked at 30 minutes postprandial after the ingestion of the pure QPM grits, and QPM grits mixture. On the other hand, peak mean blood glucose concentration was achieved at 45 postprandial after ingestion of milled rice. The computed incremental area under the glucose response curve (IAUC) level varied significantly across test food (*P* < .0379) (refer to Table 3). The IAUC of boiled rice was higher by 6.46 than that of white bread (control). Boiled rice-QPM mixture yielded lower IAUC than the control by 44.89. The pure QPM grits however, yielded the lowest IAUC relative to the control by 46.38. 

The average glycaemic index for milled rice (119.89 (SEM 22.65)) was higher while that of the pure QPM grits (80.29 (SEM 17.11)) was lower than the control food. The mixed rice-QPM grits had higher GI (91.29 (SEM 33.61)) than the pure QPM grits, but its GI value was still lower than that of the control food. The different subjects, however, exhibited varying glycaemic response to the different test foods, resulting in high standard errors. To address these high standard errors of the glycaemic response, two alternative robust statistical methods (regression analysis and bootstrap) were applied. 

Since the initial blood glucose among the subjects varied each time they consume the different test foods, a regression analysis was made with glycaemic index as the dependent variable. The initial blood glucose was considered as a regressor in addition to the dummy variables accounting for the different test foods (with the control food as the baseline). The test food-initial blood glucose interaction was also included. The resulting regression model was significant (*P* < .0177) with a coefficient of determination of 32%. The average glycaemic index was computed from the regression on the initial blood glucose taking into account its interaction with the test foods (see Table 4).

Resampling method (bootstrap) was also applied to analyze the possible bias introduced into the average glycaemic index caused by a few extreme glycaemic responses. For each test food, 500 replications were made, bias was computed, and the average glycaemic index was adjusted for the bias. The estimates are summarized in Table 4.

While the estimated average GI for the different test foods did not vary significantly across different estimation methods, the bootstrapped estimates yielded the lowest standard errors. Using the bootstrap method, the GI values of the test foods (bootstrapped) were 80.36 (SEM 14.24), 119.78 (SEM 18.81), and 93.17 (SEM 27.27) for pure QPM grits, milled rice, and rice-QPM grits mixture, respectively.

## 4. Discussion

This study showed that ingestion of pure QPM grits resulted in lower blood glucose response in healthy subjects compared to milled rice and the rice-corn grits mixture. Differences in the chemical composition and physicochemical properties of the test foods may have contributed to the differences in the glucose response observed. QPM grits have thick vitreous endosperms [14] and undergo rigorous drying in the conversion of kernels to grits that renders it difficult to gelatinize. Comparing the cooking time of the test foods, it can be seen that pure QPM grits and the rice-corn grits mixture took longer to cook than milled rice. As shown previously, milled rice had a shorter cooking time and higher volume expansion compared with brown rice. Milled rice has also been shown to have low amylograph viscosity peak and consistency, an indication that it can be easily hydrated and gelatinized during food processing [11]. 

Amylose analysis of the test foods showed that pure QPM grits and milled rice have comparable amylose contents—25.04 and 23.95 for milled rice and QPM grits, respectively. This supports the study of Panlasigui et al. [5] that foods with similar amylose may still exhibit varying rates of starch digestibility and blood glucose response.

Although the fats and proteins may lower the glucose response to a food item, the negligible amounts of these nutrients present in each test food investigated would not have strongly affected the observed glucose responses. As shown by a previous study, about 23 grams of fat is needed for fat content to significantly affect the glucose response to a food item [15]. On the other hand, 20–30 grams of protein is needed to sufficiently affect the glycaemic responses [16–18]. 

Pure QPM grits have the highest dietary fiber content (6.0 grams/100.0 grams of QPM grits) among the test foods (see Table 2). Dietary fiber may have contributed to the lower glycaemic response in the pure QPM grits. As previously investigated, varying fibre content of foods may cause fluctuations in the absorption of dietary carbohydrate and, therefore, affect the GI [15, 19]. Dietary fibre, depending on its type, acts either as a physical barrier or increases the viscosity of the mixture in the digestive tract so that digestion and absorption is slowed down [20]. Given that most foods contain more insoluble fibre, insoluble fibre was related more strongly to the GI than soluble fibre content [21]. Pure QPM grits have higher insoluble fiber content than soluble fiber [22]. 

The GI of the rice-QPM mixture was compared to its theoretical GI value computed using the GI values of the pure QPM grits and milled rice. The theoretical GI value of the rice-QPM grits mixture is 95.94 similar to the GI value (93.17) obtained in the in vivo testing, supporting the postulate that GI of mixed meals may be computed by determining the amount of total carbohydrates contributed by each food component and its corresponding GI values [20, 23]. 

In conclusion, pure QPM corn grits have a lower glycaemic response compared to milled rice and the rice-corn grits mixture, which may be related in part to differences in their dietary fibre composition and physicochemical characteristics. Pure QPM corn grits may, therefore, be a more health beneficial food for diabetic and hyperlipidemic individuals.

## Figures and Tables

**Table 1:** Characteristics of the subjects (*N* = 10).

Parameter	Baseline		Post-test	
	Mean	SEM	Mean	SEM
Age (years)	21	0.4	21	0.4
Body Weight (kg)	49.0	1.4	8.5	1.5
Height (cm)	158.9	1.4	158.9	1.4

**Table 2:** Proximate composition of the test foods.

Test Food	Moisture, %	Fat, %	Protein, %	TAC, %	Ash, %	TDF, %
Pure QPM grits	69.27	0.09	1.98	20.94	0.04	6.00
Milled rice	72.52	3.26	3.14	20.88	0.09	0.54
rice-QPM grits mixture	64.32	0.50	2.04	28.92	0.62	3.00

**Table 3:** Incremental area under the glucose response curves of the test foods (*N* = 10).

Test Food	Mean IAUC	SEM
Control bread	152.66	18.44
Pure QPM grits	106.28	11.76
Milled rice	159.12	11.72
Rice-QPM grits mixture	107.77	20.36

**Table 4:** Estimates of glycemic indices of the test foods (*N* = 10).

Test Food		Mean GI	SEM
Pure QPM grits	Normal^A^	80.29	17.11
	Controlled for Initial Glucose^B^	80.29	16.14
	Bootstrapped^C^	80.36	14.24

Milled rice	Normal^A^	119.89	22.65
	Controlled for Initial Glucose^B^	119.32	19.81
	Bootstrapped^C^	119.78	18.81

Rice-QPM grits mixture	Normal^A^	91.29	33.61
	Controlled for Initial Glucose^B^	90.92	22.08
	Bootstrapped^C^	93.17	27.27

^A^Based on the assumption that the observations are normally distributed.

^B^Based on the regression model that controls for the initial glucose level.

^C^Bias-corrected based on the bootstrap estimate of 500 replications.

## Nonstandard Abbreviations

QPM: Quality protein maize
GI: Glycaemic index
IAUC: Incremental area under the glucose response curve
TDF: Total dietary fibre
TAC: Total available carbohydrates.
